# Pectins and Olive Pectins: From Biotechnology to Human Health

**DOI:** 10.3390/biology10090860

**Published:** 2021-09-02

**Authors:** Maria C. Millan-Linares, Sergio Montserrat-de la Paz, Maria E. Martin

**Affiliations:** 1 Department of Food & Health, Instituto de la Grasa, CSIC. Ctra. de Utrera Km. 1, 41013 Seville, Spain; mcmillan@ig.csic.es; 2 Department of Medical Biochemistry, Molecular Biology, and Immunology, School of Medicine, Universidad de Sevilla, Av. Sanchez Pizjuan s/n, 41009 Seville, Spain; 3 Department of Cell Biology, Faculty of Biology, Universidad de Sevilla, Av. Reina Mercedes s/n, 41012 Seville, Spain; mariamartin@us.es

**Keywords:** pectin, polysaccharide, galacturonic acid, cell wall, by-products, bioactivity, olive

## Abstract

**Simple Summary:**

Pectins comprise complex polysaccharides rich in galacturonic acid, that exert many functions in higher plants as components of the cell walls, together with cellulose or lignin. The food industry has traditionally used pectins as an additive due to their gelling or thickening properties. Pharmaceutical research is also taking advantage of pectin bioactivity, providing evidence of the role of these polysaccharides as health promoters. Fruits and vegetables are natural sources of pectins that can be obtained as by-products during food or beverage production. In line with this, the aim of our study is gathering data on the current methods to extract pectins from fruit or vegetable wastes, optimizing yield and environmentally friendly protocols. Updated information about pectin applications in food or non-food industries are provided. We also point to olives as novel source of pectins that strengthen the evidence that this fruit is as remarkably healthy part of the Mediterranean diet. This work exhibits the need to explore natural bioactive components of our daily intake to improve our health, or prevent or treat chronical diseases present in our society.

**Abstract:**

Pectins are a component of the complex heteropolysaccharide mixture present in the cell wall of higher plants. Structurally, the pectin backbone includes galacturonic acid to which neutral sugars are attached, resulting in functional regions in which the esterification of residues is crucial. Pectins influence many physiological processes in plants and are used industrially for both food and non-food applications. Pectin-based compounds are also a promising natural source of health-beneficial bioactive molecules. The properties of pectins have generated interest in the extraction of these polysaccharides from natural sources using environmentally friendly protocols that maintain the native pectin structure. Many fruit by-products are sources of pectins; however, owing to the wide range of applications in various fields, novel plants are now being explored as potential sources. Olives, the fruit of the olive tree, are consumed as part of the healthy Mediterranean diet or processed into olive oil. Pectins from olives have recently emerged as promising compounds with health-beneficial effects. This review details the current knowledge on the structure of pectins and describes the conventional and novel techniques of pectin extraction. The versatile properties of pectins, which make them promising bioactive compounds for industry and health promotion, are also considered.

## 1. Introduction

Pectins are present in the primary cell walls and middle lamellae in higher plants within a complex heteropolysaccharide matrix, which contains up to 30% pectins together with cellulose and hemicellulose [1,2] resulting in networks due to linkages among them. Carbohydrates are the major components of the cell wall, which contain only 5–10% of proteins, including extensins and arabinogalactan proteins [3,4]; all are modified during fruit ripening. Despite the diversity of their chemical composition across species and tissues, pectins are known to play a key role in plant tissue firmness and plant development, modulating the properties of the cell wall and cell functions. In plant tissues, pectins in the middle lamella also contribute to cell-to-cell adhesion and act as a barrier against pathogens [3,5,6]. Many studies have also highlighted the interaction between pectin chains and the cellulose-hemicellulose network [1,4,7,8,9,10,11,12,13].

Pectin polysaccharides have been extensively used as a functional ingredient in the food industry and also in non-food industries during the production of cosmetics, packaging materials or pharmaceuticals. Over the last few years, several studies point to an increasing interest in pectins as health-promoting molecules for biomedical applications. Nevertheless, it is well established that pectin extraction methods strongly influence the structure and properties of these polysaccharides [1,14]. This review summarizes current knowledge concerning pectin sources and extraction protocols. Additionally, we provide evidence that olive fruits may be a promising natural source of bioactive pectic polysaccharides obtained during olive oil production, which also valorize traditional industrial by-products or wastes.

### Pectins from Olives

Cultivation of *Olea europaea* L. (the olive tree) dates back more than 7000 years [15] and is widespread, owing to the continually increasing demand for both table olives and olive oil for human consumption. Globally, the Mediterranean region is the largest cultivator of olive trees, responsible for 98% of the world’s production; moreover, the so-called “Mediterranean diet” includes olive oil, which is a remarkable healthy fat known to have cardioprotective and anticancer activity [16]. In addition, high-value compounds’ unexpected bioactivities have been identified from different parts of the olive (fruits and leaves) and in waste materials produced during olive oil extraction [17,18]. The increased attraction of renewable bioresources has stimulated research into the recovery of potential health-beneficial products from olive trees. Nevertheless, the quality of both the olive fruit and olive oil is affected by ripeness, cultivar, harvest conditions, and processing technology [15].

The olive fruit contains three different regions: the external skin or epicarp, which contains wax; a soft pulp or mesocarp; and a hard stone or endocarp. Water (50%), oil (22%), and carbohydrates (19%) are the major components, with lower proportions of cellulose (6%), proteins (1.6%), phenols (1–3%), and inorganic chemicals (1.5%) present. Minor compounds, including pectic polysaccharides, organic acids, and pigments, are also present in the olive fruit. Olive oil is considered a “functional food” as it contains oleic acid, other monounsaturated fatty acids (MUFA), phenolic compounds, and other minor bioactive molecules [15]. Phenolic compounds are already known to have remarkable health-promoting activities, and recent research into the bioactive properties of many fruits and vegetables has focused on pectins as a medicinal and therapeutic novel target [15].

## 2. Structure, Quantification, and Qualification

### 2.1. Pectins

Pectins are complex heteropolysaccharides, which include at least 17 kinds of monosaccharides and over 20 types of linkages, with a backbone of α-1,4-D-galacturonic acid (70%) in which homogalacturonan (HG), rhamnogalacturonan (RG-I and RG-II), and xylogalacturonan (XG) domains, linked by covalent or ionic interactions, can be distinguished [14,19]. Homogalacturonan linear domain monosaccharides are partially C-6 methyl-esterified and may be C-2/3 O-acetylated in some plant sources, and the degree of esterification is a parameter that affects pectin functionality [20]. This “smooth region” of HG is the most abundant pectin domain (comprising 60–65%) in plant cell pectins [19] and has been recently related to epidermal morphogenesis in plants [21]. The “hairy” regions of pectin molecules include both RG-I and RG-II, to which nonionic side chains containing many neutral sugars are attached [22]. RG-I domains include rhamnose residues in the galacturonic acid backbone with many side chains containing other neutral sugars, such as galactose or arabinose [8]. It is well established that the monosaccharide composition and architecture of both HG and RG-I domains vary significatively during plant development [23]. Only little structure variations in pectin RG-I domains have been reported in different plants [24]. RG-II is a much more complex domain, in which up to 12 types of sugar may be present, including the rarely observed apiose, xylose, or fucose [24]. Despite only being a minor region in pectin, RG-II is well preserved in different plant species and plays a key role in the cell wall structure [8,22,24]. The xylogalacturonan domain is present in many storage and reproductive plant tissues [25]. 

**Figure 1:**
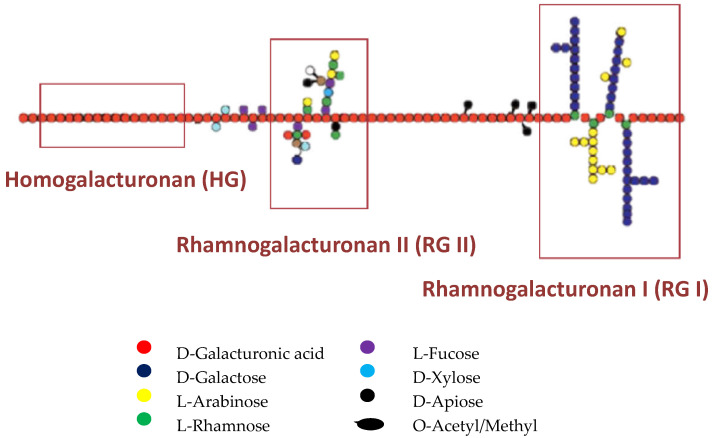
Schematic representation of the structure of pectins, showing the galacturonic acid backbone, and the homogalacturonan, rhamnogalacturonan, and xylogalacturonan regions of the molecule. Modified from [26].

Many models have been proposed to explain the macromolecular structure of pectins in which polysaccharides are covalently bound, but the precise position of the attached hairy and smooth regions is still under debate [1,2,19]. The isolation and determination of pectin components have been extensively assessed to identify the design of plant cell wall networks in which pectins are also bound to cellulose and hemicellulose (Figure 1).

### 2.2. Olive Pectins

The industrial production of olive oil generates huge quantities of a wet organic matter commonly known as olive pomace, composed of 60–70% water and containing 98% of the total phenols in the olive fruit, known for their beneficial properties for health [27]. Pectic polysaccharides comprise approximately 39% of this wet olive pomace. The degree of methyl esterification is approximately 48% and the degree of acetylation is approximately 11%. Compared to citrus commercially available low-methoxyl-pectins, olive pomace pectin extracts show a higher degree of methyl-esterification, acetylation, and total neutral sugar content, but a lower galacturonic acid percentage or molecular weight [27]. The presence of arabinan-rich pectic polysaccharides in olive pomace is notable, and its quantification is a parameter to evaluate the ripeness of the olive fruits [27]. These agricultural wastes therefore appear to be an interesting source of health-beneficial biomolecules that can be recovered to yield environmental and economic benefits [25].

Many studies have focused on the cell wall modifications of fleshy fruits during ripening. Enzymatic and non-enzymatic activities alter the polysaccharide structures of hemicellulose and pectins and may even cause variation in the textures between cultivars [28]. However, very little is currently known about these chemical modifications during modification in olive fruits [6,29,30,31,32,33]. Some studies indicate a key role for gene expression, increased enzyme activities, and the loss of neutral sugars during maturation in the solubilization and rearrangements in olive cell wall pectins [20].

## 3. Extraction

### 3.1. Pectins

Historically, pectins have been extracted from vegetables and fruits during food processing (Table 1). The by-products from juice production, such as apple pomace (14%) [34] and citrus peel (85%) [35], are the most useful sources of commercial pectins [2]. Among citrus fruits, the peels of orange [36], lemon, lime, and grapefruit [37] are rich in pectins. The wastes from tomato, carrot, and pumpkin have been used for pectin extraction. Sugar beet pulps [38], potatoes, sunflower seed heads, cocoa husks, mulberry branch barks, bean hulls, sisal wastes, watermelon rinds, pomegranate, pineapple, mango, papaya, passion fruit [39], or banana peels [40], and kiwifruit pomace, are novel sources of plant pectins [1,14,22,25,41,42,43] (Table 2).

From the raw biomass, the industrial process of extraction requires pre-extraction protocols, followed by hydrolysis and isolation of pectins and post-extraction solubilization. Pretreatment processes include drying, washing or blanching and aim to inactivate enzymes or bacteria that preserve stability of material and prevent deterioration of pectic polysaccharides [1,14,24,42].

Both single digestions and combined methods have been used extensively for pectin extraction [1,14,24,42]. Single extraction methods use acid or alkali solutions in addition to enzyme treatments to release pectins from the cell wall, where it forms complex networks with cellulose and hemicellulose. The use of chemicals is more economic than enzyme hydrolysis, although alkali extraction achieves high yields but results in environmental pollution. Acid extraction combines a high temperature and a strong mineral acid, such as hydrochloric, sulfuric, or nitric acid. Organic acids, such as citric or acetic acid, may preserve the native pectin structure compared with other acids [14]. 

**Figure 2:**
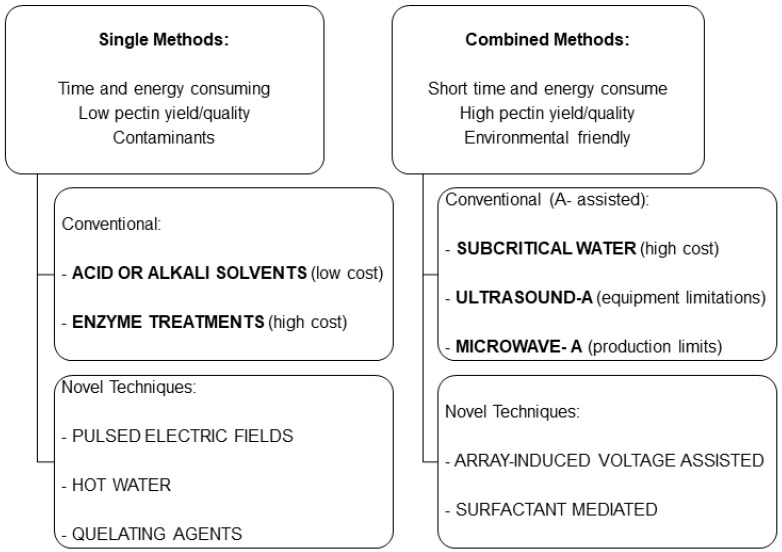
Schematic comparing the extraction methods of pectin. A-assisted techniques include additional energy supply [1,14,24,42,46].

Pulsed electric field extraction or the use of hot water or chelating agents, such as oxalate or sodium hexametaphosphate, are also single extraction methods [1,45]. A pulsed electric field applies a high voltage during a short time to a food product, increasing cell membrane permeability and facilitating bioactive molecules release [45]. Nevertheless, these protocols are time- and energy-consuming, with low extraction yields and inadequate pectin quality or functionality, as well as environmental disadvantages due to contaminants generated [14,46]. However, the structure and properties of pectins are influenced by the extraction method; thus, there is a need to find novel extraction techniques that achieve the optimal yield and quality of the by-products generated and the isolated pectic polysaccharide products [42]. Accordingly, combined techniques using subcritical water-, ultrasound-, microwave-, or ultrasonic/microwave-assisted protocols are promising approaches for pectin extraction [1]. They aim to improve the quality and the yield when extracting natural compounds from biological materials without increasing the economic or environmental impact [14,42]. Subcritical water is an alternative solvent consisting of liquid water at an elevated pressure able to achieve very high temperatures, over the boiling point, without a change of phase. Higher temperatures reduce the strength of hydrogen bonds and the energy required to disrupt complex interactions in cell walls [1,42]. Subcritical water protocols and ultrasound- or microwave-assisted methods shorten pectin extraction times and achieve high yields, although the use of subcritical water is relatively expensive [1]. Although the ultrasonic/microwave-assisted methods are limited by the equipment required, remarkable yields have been obtained. Promising results have been reported with novel combined procedures, such as array-induced-voltage-assisted extraction or surfactant-mediated pectin extraction [1] (Figure 2). Array-induced-voltage protocol applies a voltage in an acidic medium generating electromigration of charged solutes that interact with each other. Pectins can then be released from cell walls and intercellular spaces [1]. On the other hand, surfactant-mediated techniques take advantage of micelles generation at a certain surfactant concentration and the variety of interactions that micelles can stablish with pectin polysaccharides [1].

The final purification of the extracted pectins can be performed by several techniques. Precipitation of the extracted material, alone or in combination with filtration, dialysis, ionic exchange, or nitration, are some examples of accepted methods [14,24] (Table 3). As already mentioned, it is noteworthy the relationship between the complete extraction process and the chemical structure of final purified pectins.

### 3.2. Olive Pectins

At present, two-phase extraction is preferred in the olive oil industry as it reduces the consumption of water and the generation of liquid pollution. The resulting solid phase includes water and vegetable mass and is commonly known as “wet olive pomace” [15,27]. Pectins are minor compounds in the olive fruit but comprise up to 35% of the olive pomace during processing [25,27], depending on the ripening stage and other factors related to cultivar conditions and olive variety [33,47,48]. Few data available (Table 4) prevent a comprehensive understanding of the changes in the olive pulp cell wall polysaccharides during ripening in different cultivars. In general, olive maturity entails higher oil content but lower pectin content, in which molecules become more soluble and branched and exhibit a lower degree of esterification [27,48]. Pectin degradations appear to be caused by enzymatic activities, though it has also been demonstrated that new polysaccharides can be produced during ripening. 

Pectins can be extracted from olive pomace as an “alcohol-insoluble residue” (AIR), which also includes additional cell wall materials such as cellulose, hemicellulose or proteins [33] (Table 4). Conventional methods already described, such as high temperature or acid solvents, have been used extensively in extraction protocols [47,49,50]. Some data point to low molecular weight pectins as bioactive compounds and, accordingly, hydrothermal treatment has appeared as a promising technology for the production and solubilization of pectins from olive pomace, as temperature is a critical parameter for maintaining the bioactivity of pectin [51,52]. Regarding the million tons of olive pomace produced every year by the olive oil industry, this by-product appears to be a noteworthy source of bioactive molecules, including pectic polysaccharides.

## 4. Industrial Applications

### 4.1. Pectins

To isolate health-promoting pectins from plants, many strategies have recently been studied to develop functional foods [24,53,54,55]. Nevertheless, extensive in vivo research is required to confirm the bioavailability of pectin oligosaccharides in both animal and human diets; and, as already stated, food and non-food industries may need to consider that the extraction method influences both the physicochemical markers and the bioactivity of pectins [1].

Pectins have been used historically as additives in the food industry, including gelling, emulsifying, and stabilizing agents, as well as texture or thickness modulators, and fat-replacing components [22,54]. They have good biocompatibility and biodegradability, lack toxicity, and contribute to our dietary soluble fibers as no enzymatic digestion pectins occur in the human upper gut [22]. Nevertheless, some properties of pectins are strongly influenced by the number and localization of the esterified residues in the homogalacturonan region of the molecule [8]. Consequently, high-methylesterified (HM, 60–80%) and low-methylesterified (LM, 30–40%) pectins are suitable gelling agents for various products. Vegetable jellies include LM pectins, whereas other jellies, marmalade, mayonnaise, juices, or canned fish include HM pectins, which are more suitable for gelation [22,43].

The properties of pectins are also used in non-food industries, such as the pharmaceutical or cosmetics industry. As an emulsifier or thickening agent, pectins are present in cosmetic products and they are also useful as delivery vehicles for genes [50] or drugs [22,56,57,58]. Other industrial applications suggest that pectin-containing polymers are suitable for the preparation of biomaterials for various purposes [22] (Table 5).

It should be noted that not only the degree of esterification, but the pectin conformation, monosaccharide composition, and molecular weight are also strongly influenced by the extraction method [1]. Further research should be undertaken to optimize the pectin-based products of industrial interest.

### 4.2. Olive Pectins

Olive pomace polysaccharides have an 11% (acetyl)–48% (methyl) low degree of esterification, which points to the gelling potential as a food ingredient of this by-product in oil production [25]. What is more, in the presence of calcium, olive pomace pectins are able to form elastic gels more resistant to high temperatures than those commercial low-methoxyl-pectin/calcium gels [27]. The emulsifying activity of olive pomace polysaccharides has been proven compared with traditional sources of pectins [52].

## 5. Bioactivity

### 5.1. Pectins

New ventures to find natural sources of pectins in plants have the potential to expand what is known about vegetal polysaccharides as bioactive compounds that are available in large quantities but are still considered as waste. Many biomaterials are based on the pectin molecule, and many studies have assessed the efficiency of pectins as wound-healing agents [77] or in tissue engineering [22,78,79]. Pectins are a common dietary source of oligosaccharides from fruits and vegetables that are fermented in the colon by the gut microbiota. Promising activities include bactericidal, immunomodulatory, anti-inflammatory [80,81], antioxidant, cardioprotective, probiotic [82], cholesterol [83], serum glucose-reducing [84], and intestinal and obesity regulator [25,77,85] functions for pectin oligosaccharides. Moreover, low molecular weight fragments from pectins exhibit antitumoral activities [25,86,87,88,89,90]. Recent studies have also pointed to the importance of fruit and vegetables as an important source of pectin molecules containing the RG-I domain [23,91] (Table 6).

As previously stated, some health-beneficial functions of pectins are known to be strongly affected by the extraction technique, with changes in the immunomodulatory, anti-inflammatory, or probiotic activities of pectins [1]. 

### 5.2. Olive Pectins

The chemical composition of olive fruits varies depending on the cultivar, environmental conditions, and the maturation from green to black fruits. Many studies have provided data concerning olive phenols [113], but despite the importance of pectin transformation in the cell wall, there is little published research on this topic [6,15,25,33].

As already stated, the olive pomace resulting from olive oil production has been described as a valuable source of olive pectins [27]. Given the economic and environmental relevance of olive cultivars and the increasing popularity of natural, bioactive, and healthy phytochemicals, olive pectin extracts are a potential new complement for both nutrition and health improvement that support research into the composition and distribution of olives [15].

Polysaccharide-enriched extracts from olive pomace have shown health-promoting activities in in vitro experiments, including those related to antioxidant behavior and the regulation of glucose or lipid metabolism compared with commercial pectins [52]. There are promising results demonstrating the antitumoral activity of pectin extracts from olive oil by-products [90].

## 6. Conclusions

This work aims to gather current knowledge regarding pectin polysaccharides, essential components of plant cell walls that play key roles in plant development and physiology. Food and non-food industries have taken advantage of pectin bioactivity, exhibiting the need of exploring new sources of natural pectins from fruits and vegetables. Novel extraction methods, optimizing both yield and quality of pectins obtained, as well as unexplored plants for pectin recovery during food processing, require further investigation. Since pectin molecules exhibit promising health-promoting properties, further research should be undertaken to reach the goal of producing pectin bioactive components and, at the same time, valorize traditional agroindustry by-products considered as wastes. In line with this, olive fruit appears as a remarkable source of natural pectins, containing many known healthy unsaturated fatty acids from an outstanding economic impact cultivar around the Mediterranean basin. Our study points out that the challenge from now on is optimizing pectin production from industrial by-products based on novel fruits or vegetables and explore bioactivity of pectin components that may lead to nutraceuticals or functional foods able to improve our health and even prevent chronical diseases. To the best of our knowledge, our review highlights for the first time the need of research regarding olive fruit pectins as potential molecules involved in human health promotion. Although olives are known as part of the healthy Mediterranean diet due to their bioactive components, we show that only a few studies have focused on the activities of pectins from olive fruit. This study emphasizes as a novelty the importance of olives as natural sources of pectin polysaccharides in combination with the valorization of by-products or wastes from industrial processes such as olive oil production.

## Figures and Tables

**Table 1:** Pectin content of agricultural by-products [25,42,44].

Source	Total Production (Tonnes)	By-Product (% Fruit Weight)	% Pectin in By-Product
Apple waste	3.8 × 10^5^ pulp	5–10% pomace	15–21%
Lemon peel	8 × 10^4^	NA	30%
Grapefruit pomace	NA	5–10%	NA
Pomelo peel	NA	10–15%	NA
Sugar beet pulp	9.1 × 10^7^	NA	15–30%
Potato pulp	1.3 × 10^5^	NA	15%
Watermelon rind	NA	50–60%	13–30%
Mango peel	NA	15–20%	10–15%
Passion fruit peel	NA	50–60%	15–20%
Banana peel	NA	20–30%	4–6%
Olive pomace	1.6 × 10^6^	NA	34%

**Table 2:** Pectin yield from plant sources and composition [24,44].

Source	Yield of Extracted Pectin	Galacturonic Acid Content	Degree of Esterification
Apple pomace	10–20%	58–67%	52–76%
Lime peel	13–26%	91%	82%
Orange peel	24%	68%	37%
Grapefruit waste	25–30%	NA	NA
Pomelo peel	6–37%	NA	NA
Sugar beet pulp	24%	72%	28–52%
Pumpkin waste	7%	63–73%	3–18%
Carrot pomace	5–15%	62–69%	53–77%
Carrot peel	9%		
Tomato pomace	7%	78%	76–88%
Tomato peel	32%		
Watermelon rind	3–28%	68–74%	61–63%
Mango peel	5–17%	29–53%	85–88%
Passion fruit peel	8–12%	66–68%	45–60%
Banana peel	2–9%	40–71%	1–80%

NA: not available.

**Table 3:** Percentage yields of pectin extracted using several methods.

Source	Solvent Extraction	Enzyme Extraction	SWE	UAE	MAE	UMAE
Apple pomace [1,14]	3–23%	3–14%	10–16%	9%	23%	
Lime peel [44]		23%				
Orange peel [1,14,44,46]	3–23%	11%		28%	5–26%	
Grapefruit waste [1,14,46]	17–24%			3–32%		
Pomelo peel [1,14,44,46]	3%		3–19%	3–38%	0.05–29%	36%
Sugar beet pulp [14,46]				26%	5–32%	
Pumpkin waste [14,46]				22–23%	3–7%	
Carrot waste [14,46]	5–15%			27–35%		
Tomato waste [14,46]	9–19%			15–36%		
Watermelon rind [14,44]					13–24%	
Mango peel [44,46]	5%			8–17%		
Passion fruit peel [14,44,46]	5–14%	3–26%		7–13%	30%	
Banana peel [14,44,46]	5–12%			21%	1–2%	

SWE: Subcritical water extraction. UAE: Ultrasound-assisted extraction. MAE: Microwave-assisted extraction. UMAE: Ultrasonic/microwave-assisted extraction.

**Table 4:** Oil content, AIR (alcohol insoluble residue) yield, and pectin degree of methyl esterification from several olive cultivars at different ripening stages [33].

Cultivar	Maturity Stage	Oil Content (g/100 g) DW	AIR (g/100 g)	Degree of Esterification
‘Arbequina’	Green	39.3	7.8	56.9%
	Turning	43.3	7.6	49.8%
	Ripe	52.2	3.6	64.8%
‘Argudell’	Green	39.8	12.3	77.9%
	Turning	48.0	11.4	93.1%
	Ripe	50.1	8.4	78.4%
‘Empeltre’	Green	45.9	5.1	88.5%
	Turning	45.5	3.8	48.9%
	Ripe	56.1	6.4	31.9%
‘Farga’	Green	36.4	8.6	52.1%
	Turning	40.9	5.0	58.2%
	Ripe	51.2	4.0	71.2%
‘Manzanilla’	Green	45.0	4.0	62.4%
	Ripe	50.6	3.0	67.5%
‘Marfil’	Green	46.1	7.93	49.8%
	Ripe	34.2	3.5	50.9%
‘Morrut’	Green	27.0	15.2	70.0%
	Turning	37.2	12.7	76.6%
	Ripe	45.0	6.2	74.3%
‘Picual’	Green	35.6	8.4	58.3%
	Turning	48.6	11.6	68.1%
	Ripe	55.4	7.3	76.5%
‘Sevillenca’	Green	43.8	10.1	62.7%
	Ripe	57.0	9.7	67.5%

**Table 5:** Use of pectins and pectin combinations in food and non-food industry [44,54,59].

	Use in Food Industry	Use in Non-Food Industry
Citrus [56,60,61,62,63]	Antimicrobial, gelling, and thickening	Disinfection of medical devices, genes, drug delivery, and gelling/thickening agent
Lemon peel [64]		Packaging material
Pineapple peel [65]	Inhibit lipid oxidation	
Tomato peel [66]	Corrosion inhibitor	
Grapefruit peel [37,67,68]	Gelling agent, lipid digestibility	Wastewater treatment
Fig skin [69]	Anti-radical/oxidant	
Sugar beet pulp [70]		Drug delivery
Durian rind [71]		Wastewater treatment
Jackfruit peel [72]	Antioxidant	
Sunflower head [73]	Reduce lipid uptake	
Carrot waste [74,75]	Antioxidant	
Dragon fruit peel [76]	Antioxidant	

**Table 6:** Bioactivity of pectins extracted from various sources [25,44,46,59].

Source	Application
Orange peel	Prebiotic effect [92,93]
Sugar beet pulp	Prebiotic effect [94,95,96]
	Anti-inflammatory [95]
	Antitumoral [97]
Lemon peel	Prebiotic effect [94]
Apple pomace	Prebiotic effect [98]
Citrus	Anti-diabetic [99]
	Lipid digestibility [100]
	Antitumoral [101,102,103,104,105,106,107]
Banana passion fruit waste	Lipid digestibility [100]
Pumpkin waste	Antitumoral [108]
Fig skin	Antitumoral [69]
Grapefruit peel	Antioxidant [37]
Bergamot peel	Prebiotic effect [109]
Mangosteen rind	Antioxidant [110]
Gabiroba pulp	Antitumoral [111]
Dragonfruit peel	Hypolipidemic agent [112]

## Data Availability

Not applicable.
